# Power controllable gain switched fiber laser at ~ 3 μm and ~ 2.1 μm

**DOI:** 10.1038/s41598-020-80238-9

**Published:** 2021-01-13

**Authors:** Yiwen Shi, Jianfeng Li, Chendong Lai, Hanlin Peng, Chen Zhu, Yong Liu

**Affiliations:** 1 State Key Laboratory of Electronic Thin Films and Integrated Devices, School of Optoelectronic Science and Engineering, University of Electronic Science and Technology of China (UESTC), Chengdu, 610054 China; 2 Science and Technology on Solid-State Laser Laboratory, 11th Research Institute of China Electronics Technology Group Corporation, Beijing, 100015 China

**Keywords:** Optics and photonics, Lasers, LEDs and light sources

## Abstract

Based on a hybrid pumping method consisting of a 1150 nm continuous-wave pump source and a 1950 nm pulsed pump source, we demonstrate a power controllable gain-switched fiber laser in dual wavebands at ~ 3 μm and ~ 2.1 μm. Different pumping schemes for pumping a Ho^3+^-doped ZBLAN fiber are studied. Using only the 1950 nm pulsed pump source, ~ 2.1 μm gain-switched pulses with single and double pulses are obtained separately at different pump powers. This phenomenon indicates that the 1950 nm pulsed pump source acts as a modulator to trigger different states of the ~ 2.1 μm pulses. Moreover, by fixing the 1150 nm pump power at 3.259 W and adjusting the 1950 nm pump power, the output power of the ~ 2.1 μm gain-switched pulsed laser is flexibly controlled while the ~ 3 μm laser power is almost unchanged, inducing the maximum output powers of 167.96 mW and 260.27 mW at 2910.16 nm and 2061.65 nm, respectively. These results suggest that the comparatively low power of the ~ 2.1 μm gain-switched pulsed laser in dual-waveband laser can be efficiently overcome by reasonably controlling the 1950 nm pump power.

## Introduction

The mid-infrared (MIR) spectral region is of interest to researchers studying chemical sensing, laser microsurgery and spectroscopy, because strong fundamental molecular vibrational absorption bands^1^ and the 3 ~ 5 μm atmospheric transmission window^2^ are located in this wavelength region. MIR pulsed fiber lasers^3–5^ have been rapidly developed thanks to the matured fabrication of rare earth-doped fluoride fibers, which can be used to generate stable, highly efficient and powerful MIR pulsed lasers with excellent beam quality. In recent years, the majority of demonstrations of MIR pulsed fiber lasers featured the single wavebands of ~ 2 μm, ~ 3 μm or ~ 3.5 μm and broadband MIR sources around ~ 3 μm by using different gain media of Tm^3+^-, Ho^3+^-, Er^3+^-doped fibers^6–14^, respectively. Alternatively, dual-waveband pulsed fiber lasers operating at ~ 2.1 μm and ~ 3 μm can be obtained simultaneously from a Ho^3+^-doped fluoride fiber laser. These lasers have some advantages for specialist applications in medicine and material processing. For medicine, a dual-waveband pulsed laser could be used to ablate soft tissue, such as skin, subcutaneous tissue, muscle, etc. The absorption peak wavelengths of the combination vibration of the O–H bond in water and fundamental stretching vibration of O–H are respectively in ~ 2 μm and ~ 3 μm^15^, allowing ablation with low thermal damage^16–18^. For material processing, a ~ 2.1 μm pulsed laser with high peak power can efficiently reduce the generation of carbonization and residue around the local heat zone when removing plastic materials in laser cutting, marking and drilling^19^. While a ~ 3 μm pulsed laser with short pulse duration could serve the fields of mid-infrared resonant ablation of the organic material^20^.

Demonstrations of dual-waveband mid-infrared pulsed lasers using holmium fluoride fiber have been studied^7,21–24^, owing to the transitions of the ^5^I_6_ → ^5^I_7_ and ^5^I_7_ → ^5^I_8_ which realize the emissions of ~ 3 μm and ~ 2.1 μm simultaneously. Among these studies, methods such as active/passive Q-switching and gain-switching have been adopted^25–27^. Using a TeO_2_ acousto-optic modulator (AOM), Li et al*.* demonstrated the first pulsed cascade laser at 3.005 μm and 2.074 μm—the first Q-switched laser operating beyond 3 μm^21^. They also presented a new gain-switching process in which Q-switching could induce gain-switching in an active laser cavity at dual wavebands^22^. Subsequent work^7^ sought to simplify the structure of the laser cavity by employing a specially designed semiconductor saturable absorber mirror (SESAM) as a passive modulator to directly realize the passively Q-switched induced gain-switched dual-waveband pulsed fiber laser. Compared with Q-switching, any intracavity modulator is unnecessary for gain-switching which is characterized usually by rectangular modulation function of pump^28–30^ or pulsed pump^31–33^. Gain-switching makes the laser architecture simpler and avoids extra loss induced by active/passive modulators. Luo et al*.* reported the first gain-switching induced gain-switched dual-waveband mid-infrared pulsed laser by employing 1150 nm continuous-wave (CW) and pulsed laser diodes (LDs). The maximum output powers were 262.14 mW and 75.23 mW at the center wavelengths of 2928.5 nm and 2068 nm, respectively^23^. Recently, we realized a gain-switched dual-waveband pulsed laser based on hybrid pumping^24^. By controlling the pump ratio of the 1950-nm pulsed laser and 1150-nm CW laser, the maximum output powers of 136.6 mW and 26 mW at 2971.9 nm and 2062.3 nm were achieved, respectively. The output power of the ~ 2.1 μm emission is much lower than that of the ~ 3 μm emission in the above publications, resulting from cascading induced high ~ 2.1 μm threshold^7^ or unsuitable hybrid pump power ratio for scaling ~ 2.1 μm power in dual-waveband pulsed laser.

In this paper, we report a power controllable gain-switched dual-waveband Ho^3+^-doped ZBLAN fiber laser. The maximum output power of the laser at ~ 3 μm is 167.96 mW and at ~ 2.1 μm is 260.27 mW, overcoming the maximum ~ 2.1 μm power limit of previous publications. Red and blue shifting of spectra with this hybrid pumping scheme are observed, in which the mechanisms are analyzed. The output characteristics of the Ho^3+^-doped ZBLAN fiber laser in different pumping schemes are also studied in detail.

## Results and discussion

A setup of the power controllable gain-switched dual-waveband fiber laser and a simplified energy level diagram of this laser system are depicted in the Methods section. In this section, we firstly studied the performances of the output laser in different pumping schemes, i.e., while pumping with only the 1150 nm CW pump source, only the 1950 nm pulsed pump source, and variable 1150 nm CW pump source with the maximum 1950 nm pulsed pump power.

When pumping with only the 1150 nm CW pump source, only the ~ 3 μm CW laser was observed because the number of Ho^3+^ ions released from the ~ 3 μm transition to the ^5^I_7_ level was not high enough to realize a population inversion for the ~ 2.1 μm transition. When pumping with only the 1950 nm pulsed pump source, only the ~ 2.1 μm gain-switched pulsed laser was achieved, resulting from the population in the ^5^I_7_ level being periodically modulated by the 1950 nm pulsed pump source^24^. When pumping with the variable 1150 nm CW pump source at the maximum 1950 nm pulsed pump power, the pulse states at ~ 2.1 μm changed from multiple pulses to single pulse until the ~ 2.1 μm pulse disappeared, which is similar to the phenomena reported in our previous publication^24^.

**Figure 1:**
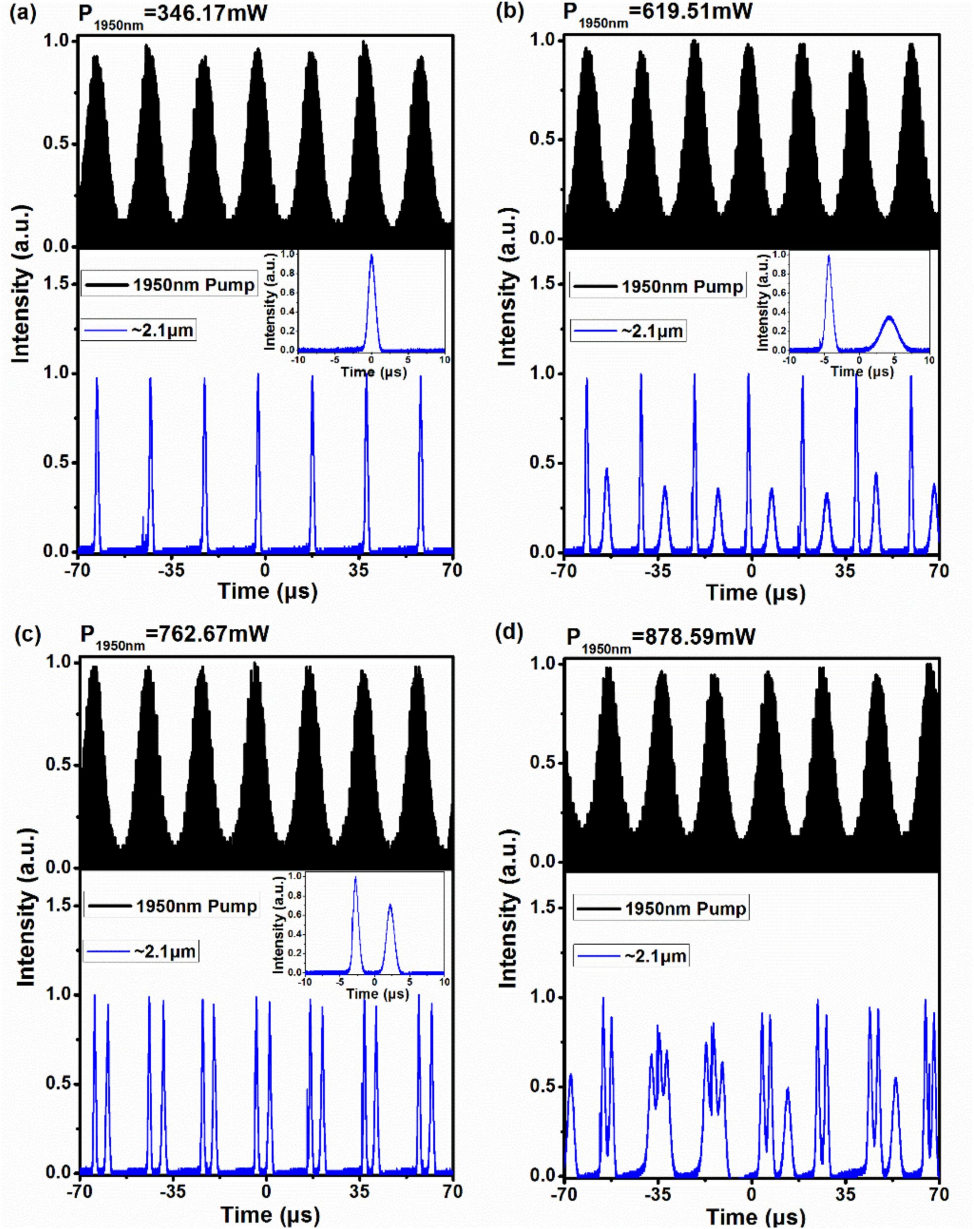
Temporal pulse trains and pulse profile (inset) of the ~ 2.1 μm gain-switching at the 1950 nm pump power of (**a**) 346.17 mW and (**b**) 619.51 mW; (**c**) 762.67 mW, (**d**) 878.59 mW without the 1150 nm CW pump source.

Figure 1 illustrates the varying pulse trains of the ~ 2.1 μm gain-switched pulsed laser at different 1950 nm pump powers without the 1150 nm CW pump source. The pump powers mentioned below refer to the average power launched into the gain fiber. With the pump power of 346.17 mW, the ~ 2.1 μm gain-switched pulsed laser had a repetition rate of 50 kHz, the same repetition rate as the 1950 nm pulsed pump source, as shown in Fig. 1a. When the 1950 nm pump power was increased to 619.51 mW, a sub pulse with low peak power was observed along with the main-peak pulse of the ~ 2.1 μm gain-switched pulsed laser, as shown in Fig. 1b. This behavior was observed because the periodic balance between the accumulation and consumption of the population in the ^5^I_7_ level was broken for generating one pulse in one pumped period. Once the 1950 nm pump power reached 762.67 mW, the stable ~ 2.1 μm double-pulse gain-switched laser was obtained with two pulses of the same amplitude in one pumped period, as shown in Fig. 1c. This is because that the population in the ^5^I_7_ level can still be accumulated to release the second gain-switched pulse with the help of the residual 1950 nm pump energy after the first gain-switched pulse has been generated. Such phenomenon is familiar to the multi-pulse laser which has been observed generally in gain-switched fiber lasers^33–35^. If increasing the 1950 nm pump power over 878.59 mW, the ~ 2.1 μm gain-switched pulsed laser was in a chaotic-oscillation state with irregularly spiked pulses, as shown in Fig. 1d. A stable ~ 2.1 μm gain-switched pulsed laser with three or more pulses in one pumped period was not observed which was probably limited by the available maximum pump power of 975.36 mW.

**Figure 2:**
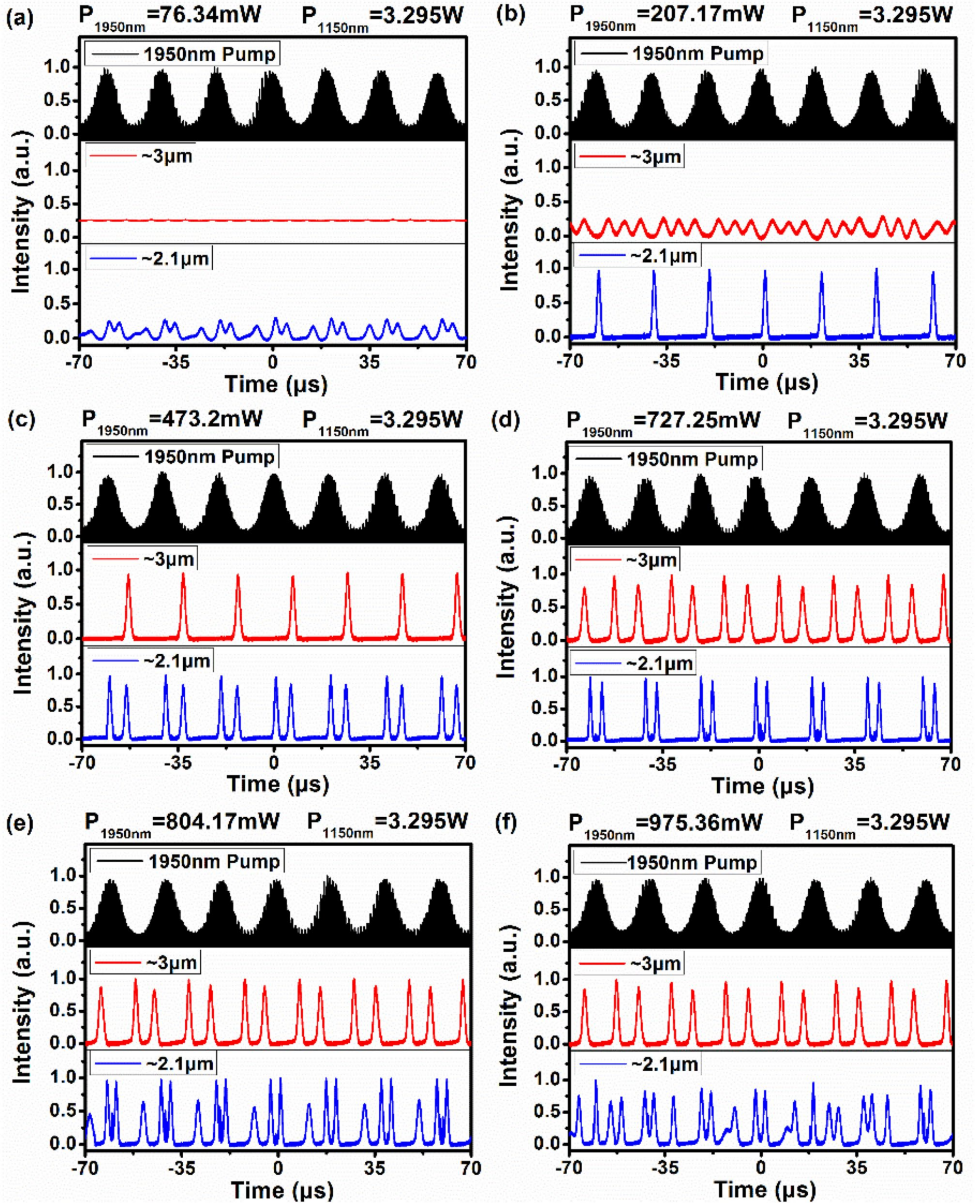
The temporal pulse evolution of dual-waveband gain-switching at the 1950 nm pump power of (**a**) 76.34 mW and (**b**) 207.17 mW; (**c**) 473.2 mW, (**d**) 727.25 mW, (**e**) 804.17 mW, (**f**) 975.36 mW with the 1150 nm pump power fixed at 3.295 W.

The output characteristics of the power controllable gain-switched laser were also investigated for varying 1950 nm pump power while the 1150 nm pump power was fixed at 3.295 W. When the 1950 nm pump power was increased to 76.34 mW, the ~ 3 μm laser operated in a CW state, while the ~ 2.1 μm laser started chaotic oscillation with low-amplitude fluctuation, as shown in Fig. 2a. Further increasing the 1950 nm pump power to 207.17 mW, the ~ 2.1 μm gain-switched pulsed laser was firstly observed, with the same repetition rate of the 1950 nm pulsed laser. Simultaneously, the ~ 3 μm laser switched to the chaotic oscillator state, as shown in Fig. 2b, resulting from the accumulated population in the ^5^I_7_ level being modulated by the 1950 nm pulsed pump source which affected the ~ 3 μm transition. Once the 1950 nm pump power exceeded 473.2 mW, the ~ 2.1 μm double-pulse gain-switched laser with two different pulse amplitudes was achieved in each 1950 nm pump pulse period, accompanied with the ~ 3 μm gain-switched pulsed laser at the repetition rate of 50 kHz, as shown in Fig. 2c. It should be noted that there is another reason for generating ~ 2.1 μm double-pulse gain-switched laser excepting the effect of the modulation of the 1950 nm pulsed laser described before. The ~ 3 μm transition can release some ions into the ^5^I_7_ level to enable the generation of the second gain-switched pulse at ~ 2.1 μm as well. Additionally, for the ~ 3 μm transition, the Ho^3+^ ions in the ^5^I_6_ level continuously fill the gap in the ^5^I_7_ level created by the transition of ~ 2.1 μm, which induces pulsed operation at ~ 3 μm. However, single-pulse states of the ~ 2.1 µm and ~ 3 µm lasers were not obtained, indicating that the energy or power of the ~ 2.1 µm single pulse could not induce the generation of the ~ 3 µm single pulse. As can be seen from Fig. 2d, the double-pulse gain-switching was simultaneously generated at ~ 3 μm and ~ 2.1 μm when the 1950 nm pump power was increased to 727.25 mW. With the further increase of the 1950 nm pump power to 804.17 mW, the ~ 3 μm gain-switched pulsed laser maintained at the double-pulse operation state, while the ~ 2.1 μm gain-switched pulsed laser generated three pulses in each 1950 nm pump pulse period, as shown in Fig. 2e. Figure 2f shows that once the 1950 nm pump power reached 975.36 mW, the chaotic-oscillation state of the ~ 2.1 μm gain-switched laser reoccurred along with the stable ~ 3 μm double-pulse gain-switched laser.

**Figure 3:**
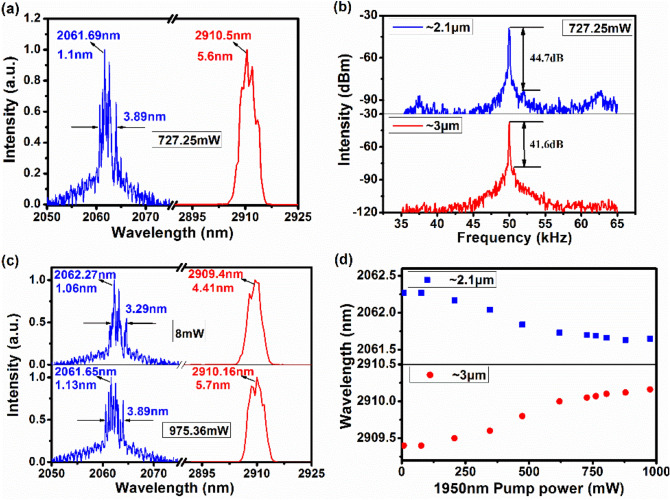
(**a**) The optical spectra and (**b**) the RF spectra of the dual-waveband gain-switching at the 1950 nm pump power of 727.25 mW, (**c**) the optical spectra of the dual-waveband lasers at the 1950 nm pump power of 8 mW and 975.36 mW, (**d**) the center wavelengths of the ~ 3 μm and ~ 2.1 μm lasers as a function of the 1950 nm pump power with the 1150 nm pump power fixed at 3.295 W.

The optical spectra of the dual-waveband gain-switched pulsed laser at ~ 3 μm and ~ 2.1 μm were measured at the 1950 nm pump power of 727.25 mW, as shown in Fig. 3a. The peak center wavelength of the ~ 2.1 μm gain-switching is 2061.69 nm with a full width at half maximum (FWHM) bandwidth of 1.1 nm, while the ~ 3 μm gain-switching has a peak wavelength of 2910.5 nm and a FWHM bandwidth of 5.6 nm. The ~ 3 µm pulsed laser operated in a single wavelength with multiple peaks while multiple wavelengths were involved in the ~ 2.1 µm optical spectrum with a spectral FWHM bandwidth of 3.89 nm measured at half of maximum intensity level. However, in our experiment, we did not find any connection between the two spectra’s structures. The mechanism is not clear and needs to be further studied.

To evaluate the stabilities of the gain-switched pulse trains at ~ 2.1 µm and ~ 3 µm, the radio frequency (RF) spectra were measured at the 1950 nm pump power of 727.25 mW, as shown in Fig. 3b. The operated central frequencies of the ~ 2.1 µm and ~ 3 µm pulses are both at 50 kHz with corresponding signal-to-noise ratios (SNRs) of 44.7 dB and 41.6 dB, respectively. This indicates that the two lasers were in stable gain-switched states. Note that the SNR of the ~ 2.1 µm pulse is slightly higher than that of the ~ 3 µm pulse due to the wavelength of the modulated pulsed pump source at 1950 nm. Compared to the ~ 3 µm pulse, the 1950 nm pump source has a stronger modulation effect on the ~ 2.1 µm pulse, which can be seen from the order of the pulse generations at ~ 2.1 µm and ~ 3 µm in Fig. 2b.

Figure 3c shows the optical spectra of the dual-waveband gain-switched pulsed laser at the 1950 nm pump power of 8 mW and 975.36 mW. The corresponding peak center wavelengths of the ~ 2.1 μm gain-switching are 2062.27 nm and 2061.65 nm with FWHM bandwidths of 1.06 nm and 1.13 nm, respectively. The spectral FWHM bandwidths measured at half of maximum intensity level of the ~ 2.1 μm multiple wavelengths are 3.29 nm and 3.89 nm, respectively. Meanwhile, the ~ 3 μm gain-switching has peak wavelengths of 2909.4 nm and 2910.16 nm with FWHMs of 4.41 nm and 5.7 nm, respectively. Combining with the spectra of the dual-waveband gain-switched laser at the 1950 nm pump power of 727.25 mW, as shown in Fig. 3a, the ~ 3 µm optical spectrum has multiple peaks while multiple wavelengths are involved in the ~ 2.1 µm optical spectrum, as a result of intra-cavity mode distribution and mode competition. Moreover, the shapes of spectra and the positions of the spectral peaks at both wavelength region are consistent after many measurements, indicating that they remain stable during operation.

Figure 3d shows the peak center wavelengths of the ~ 3 μm and ~ 2.1 μm lasers as a function of the 1950 nm pump power with the 1150 nm pump power fixed at 3.295 W. It is noted that the peak center wavelengths of the ~ 2.1 μm and ~ 3 μm lasers slightly decreases and increases with increasing the 1950 nm pump power, corresponding to blue and red shifting of the spectra, respectively. Such red (blue) shifting of spectrum is equivalent to a decrease (increase) in wave frequency and photon energy. The corresponding wavelengths blue- and red-shifted from 2062.27 nm to 2061.65 nm and from 2909.4 nm to 2910.16 nm with increasing the 1950 nm pump power from 8 mW to 975.36 mW, respectively. This is because the initial and terminal Stark manifolds of the ^5^I_7_ → ^5^I_8_ and ^5^I_6_ → ^5^I_7_ transitions are upshifted, as induced by the accumulated population in the ^5^I_7_ level when the pump power is increased, hence leading to shorter and longer wavelength emissions. These phenomena of red and blue shifting coincide with the processes of laser transitions and have been theoretically and experimentally verified in Er^3+^- and Ho^3+^-doped fluoride fiber lasers^36–40^. Note that the wavelengths could be adjusted by changing the pump power albeit with the narrow adjustable ranges of 0.62 nm and 0.76 nm for the ~ 2.1 µm and ~ 3 µm pulses, respectively. However, the narrow adjustable ranges are mainly limited by the pump power of our pump source.

**Figure 4:**
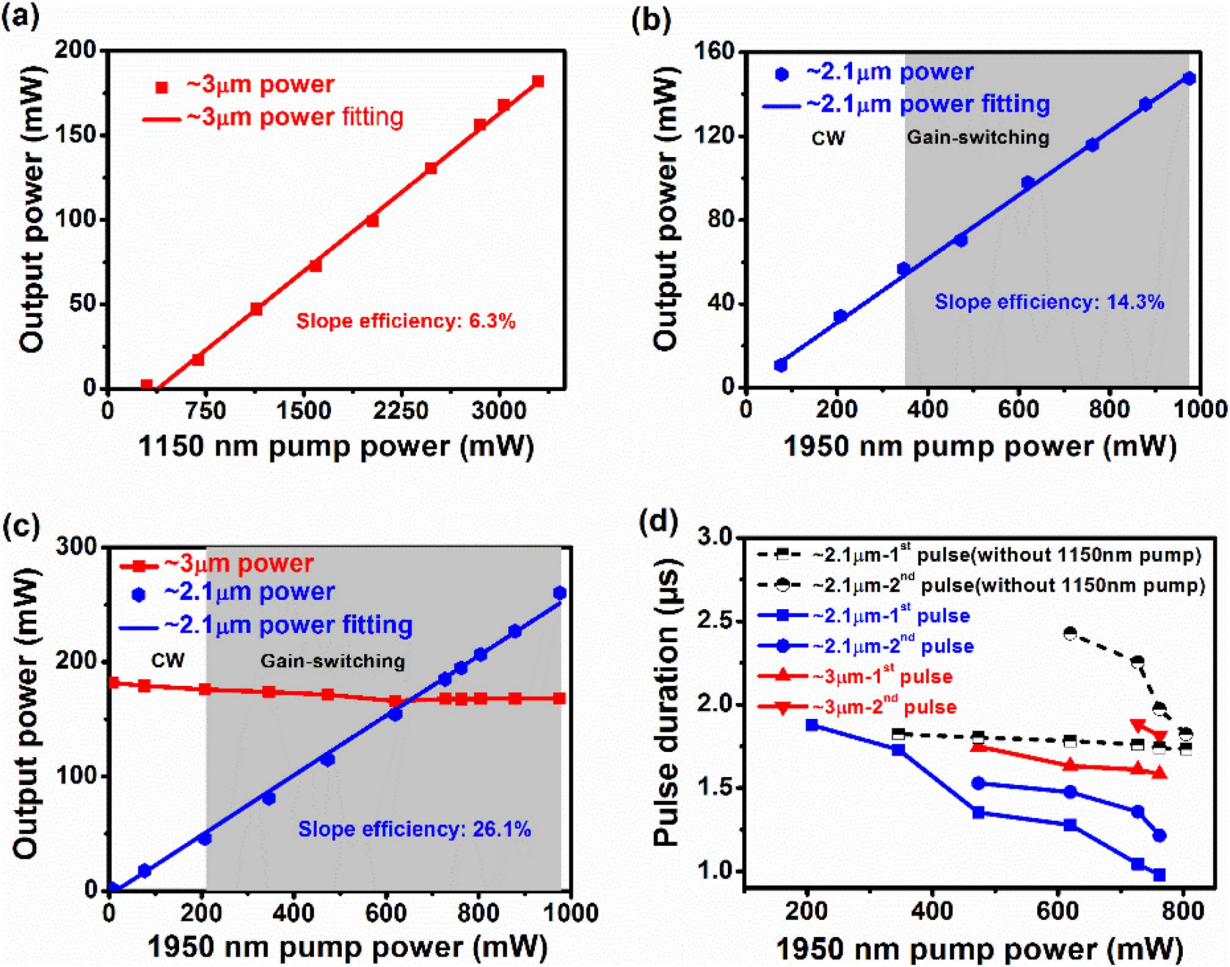
(**a**) ~ 3 μm CW output power with CW pump power at 1150 nm, without 1950 nm pulsed pump. (**b**) ~ 2.1 μm output power with pulsed pump power at 1950 nm, without 1150 nm CW pump. (**c**) ~ 3 μm and ~ 2.1 μm output power with pulsed pump power at 1950 nm and fixed pump power of 3.295 W at 1150 nm. (**d**) Variations of pulse duration at ~ 3 μm and ~ 2.1 μm in different pumping schemes.

The output powers of the Ho^3+^-doped ZBLAN fiber laser in different pumping schemes are depicted in Fig. 4a–c. Figure 4a shows the output power of the ~ 3 μm CW laser as a function of the 1150 nm pump power. It is observed that the output power increases almost linearly with the increased 1150 nm pump power at a slope efficiency of 6.3%. The maximum output power of the ~ 3 μm CW laser is 181.85 mW at the 1150 nm pump power of 3.295 W. For the case of using only the 1950 nm pulsed pump source, the output power of the ~ 2.1 μm laser has the same variation trend as the ~ 3 μm CW laser with increasing the 1950 nm pump power at a slope efficiency of 14.3%, as shown in Fig. 4b. The output power of the ~ 2.1 μm gain-switched pulsed laser increases from 56.72 to 147.63 mW with increasing the 1950 nm pump power from 346.17 to 975.36 mW. Figure 4c shows the output power of the dual-waveband fiber laser as a function of the 1950 nm pump power when the 1150 nm pump power was fixed at 3.295 W. It is seen that the output power of the ~ 3 μm laser slightly decreases from 181.85 to 165.82 mW with increasing the 1950 nm pump power from 0 to 619.5 mW and then maintains at ~ 167.7 mW. The results indicate that the output power of the ~ 3 μm laser is mainly dependent on the 1150 nm pump power. The output power of the ~ 2.1 μm laser increases almost linearly from 1.59 to 260.27 mW with increasing the 1950 nm pump power from 8 to 975.36 mW. Note that the corresponding slope efficiency is 26.1%, which is almost twice as the case of using only the 1950 nm pulsed pump source. The slope efficiencies mentioned above were calculated based on the launched pump power of the 1950 nm pump source (P_1950nm launched_). The calculated slope efficiency at ~ 2.1 μm in the hybrid pumping scheme is approximately 29.69% with respect to the absorbed power of the 1950 nm pump source (P_1950nm absorbed_). Moreover, the output power of the ~ 2.1 μm gain-switched pulsed laser exceeds that of the ~ 3 μm gain-switched pulsed laser when the 1950 nm pump power is beyond 727.25 mW, which has never been observed in previous reports. The grey regions in Fig. 4b,c represent the lasers operating in gain-switched states. Compared to using only the 1950 nm pulsed pump source, the threshold of the ~ 2.1 μm gain-switched pulsed laser is reduced from 346.17 to 207.17 mW in the hybrid pumping scheme.

Figure 4d plots the variations of pulse duration of single- and double-pulse gain-switching in different pumping schemes. The black marks represent the variations of the ~ 2.1 μm pulse duration when pumping with only the 1950 nm pulsed pump. Initially, there existed only ~ 2.1 μm single pulse with the pulse duration range of 1.824 μs-1.783 μs at the 1950 nm pump power range of 346.17 mW-619.5 mW. When further increasing the 1950 nm pump power, the ~ 2.1 μm double pulses were observed. The pulse durations of the first and second pulses decrease from 1.783 to 1.733 μs and 2.426 to 1.822 μs, respectively, with the increased 1950 nm pump power from 619.5 to 804.17 mW. The blue and red marks represent the variations of the ~ 2.1 μm and ~ 3 μm pulse durations at changeable 1950 nm pump power with the fixed pump power of 3.295 W at 1150 nm. The ~ 2.1 μm single pulse was generated firstly and the pulse duration decreases from 1.878 to 1.353 μs when the 1950 nm pump power is increased from 207.17 to 473.2 mW. Subsequently, the ~ 2.1 μm double pulses and ~ 3 μm single pulse were obtained simultaneously. The corresponding pulse durations of the first and second pulses of the ~ 2.1 μm double pulses decrease from 1.353 to 0.978 μs and 1.529 to 1.215 μs, respectively, with the increased 1950 nm pump power from 473.2 to 762 mW. The pulse duration of the ~ 3 μm single pulse decreases from 1.749 to 1.61 μs when increasing the 1950 nm pump power from 473.2 to 727.25 mW. Finally, the ~ 3 μm double pulses appeared at the 1950 nm pump power of 727.25 mW. The pulse durations of the first and second pulses decrease from 1.61 to 1.583 μs and 1.881 to 1.813 μs, respectively, with the increased 1950 nm pump power from 727.25 to 762 mW. Compared to using only the 1950 nm pump source, the pulse durations of ~ 2.1 μm gain-switching are narrower in the hybrid pumping scheme with the help of the 1150 nm pump source.

In our experiments, the Ho^3+^-doped ZBLAN fiber laser in the hybrid pumping scheme is used to obtain a power controllable gain-switched pulsed laser at ~ 3 μm and ~ 2.1 μm. The output powers and pulse states are key parameters associated with practical applications. In our case, the maximum output powers of the ~ 3 μm and ~ 2.1 μm gain-switched lasers are 167.96 mW and 260.27 mW, respectively, which are much lower than some results obtained in single-wavelength operation at ~ 3 μm and ~ 2.1 μm^41,42^. A limiting factor is the low pump light absorption in our Ho^3+^-doped ZBLAN fiber with nonoptimized length and concentration due to the available experimental equipment in our lab. Thus, selecting a longer fiber with a low-doping concentration of Ho^3+^ ions, or a high-doping holmium fiber with an appropriate length could improve the output powers, because it would be beneficial to provide > 95% pump absorption efficiencies at both 1150 nm and 1950 nm. To scale the output powers, designing a multi-step amplified all-fiber laser cavity with high-power pump sources is a promising option for future work.

The multi-pulse dual-waveband gain-switched laser was obtained at the maximum pump power in our system. However, the single-pulse dual-waveband laser at both ~ 2.1 μm and ~ 3 μm was not observed. Considering the requirements of some practical applications, there are two methods to realize this kind of single-pulse laser. One is to select an appropriate gain fiber, another is to set up other cavity configurations. For the former method, selecting a long gain fiber with low-dopant concentration could ensure the pump absorption efficiencies exceed 95% at 1150 nm and 1950 nm. Meanwhile, the pump powers of the two pump sources should be controlled carefully so that the ~ 2.1 μm single pulse could induce the generation of the ~ 3 μm single pulse. For the latter method, a single-wavelength pulsed pump source at 1150 nm can be used to obtain a cascade single-pulse dual-waveband laser^23^. Alternatively, introducing a TeO_2_ acousto-optic modulator as an active modulator^21^ or a passive modulator based on a saturable absorber^7^ into the laser cavity could achieve such a single-pulse laser.

The shortest pulse duration we obtained is in the order of microseconds, which is limited by our nonoptimized resonator configuration. Based on previous experiments^33,46^, the pulse duration in gain switching is mainly affected by pump parameters (such as pump pulse energy, pump frequency and pump pulse duration) and cavity length. In our case, using a pure high-energy Q-switched laser at 1950 nm with low frequency and short pulse duration could be benefit for achieving shorter pulses. The cavity length is positively correlated to pulse duration and should be shortened to narrow the pulse duration. However, we expect to use a long fiber with low-dopant concentration because of ensuring the fiber absorbs enough pump energy. In future, developing an all-fiber laser configuration with all-fiber devices could be an effective method to remove the free-space length of the laser cavity in our case.

To date, the laser operated in different attractors is an area to have received research attention. The behavior of the attractors can be explained in more detail and with discussion of nonlinear laser dynamics in^43–45^. This is of relevance for sensitive fiber lasers under external perturbation and their technological applications. However, the works of dual-waveband fiber lasers at ~ 2.1 μm and ~ 3 μm and longer mid-infrared wavelength fiber lasers have not yet been reported and should be further studied in the near future.

## Conclusions

In conclusion, we have demonstrated a ~ 3 μm and ~ 2.1 μm power controllable gain-switched pulsed fiber laser. The switchable single-/multi-pulse laser of single-/dual-waveband gain-switching was realized by controlling the 1950 nm pump power in different pumping schemes. When the laser was operated in the hybrid pumping scheme, red and blue shifting of spectra occurred at ~ 3 μm (shifting from 2909.4 to 2910.16 nm) and ~ 2.1 μm (shifting from 2062.27 to 2061.65 nm) emissions, respectively. By increasing the 1950 nm pump power to 975.36 mW with the 1150 nm pump power fixed at 3.295 W, the maximum output power of 260.27 mW was obtained at ~ 2.1 μm gain-switched pulsed laser, accompanied by the ~ 3 μm gain-switched pulsed laser at 167.96-mW output power. Our results open a new way for achieving a high output-power ~ 2.1 μm pulsed laser in a dual-waveband pulsed laser which could be applied to fields such as laser surgery and material processing.

## Methods

### Experimental setup of the power controllable gain-switched fiber laser

**Figure 5:**
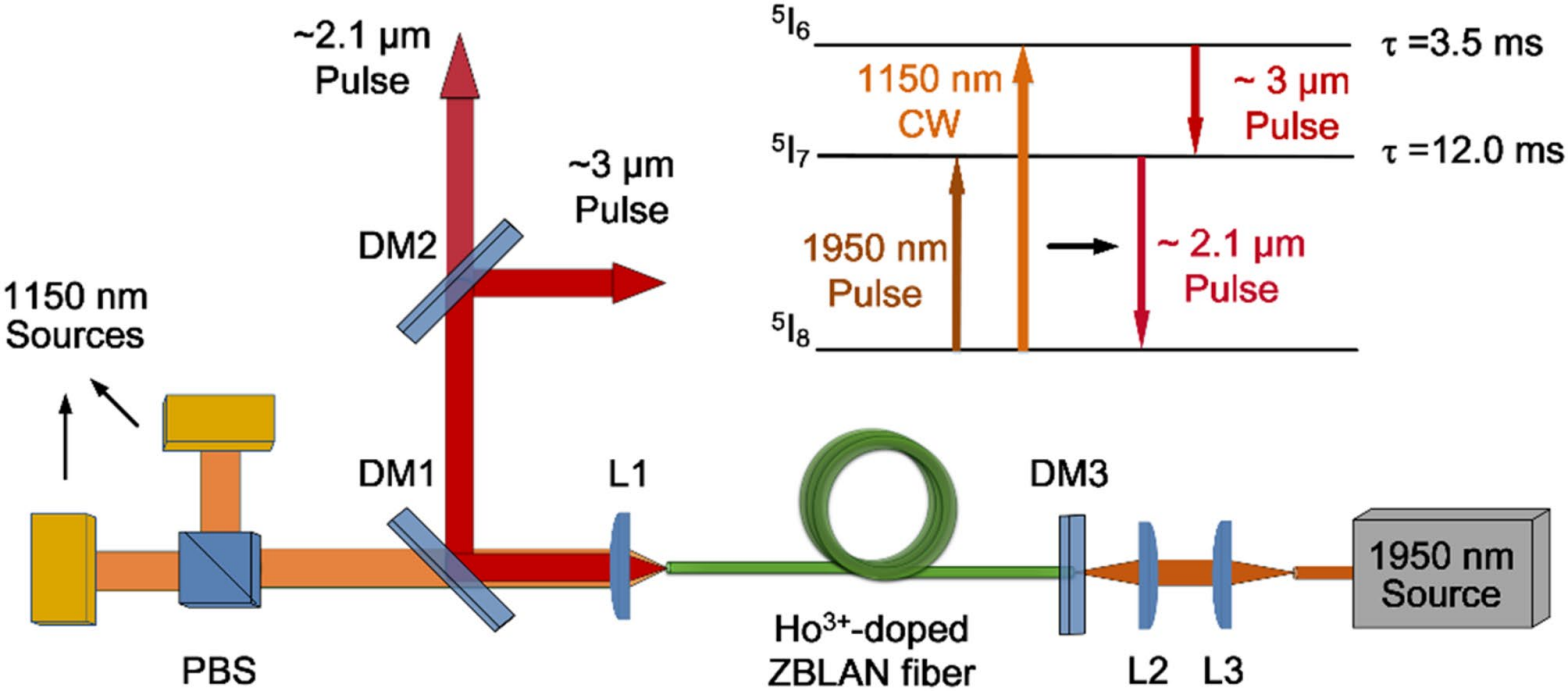
Experimental setup of the power controllable gain-switched dual-waveband Ho^3+^-doped ZBLAN fiber laser. Included is a simplified energy level diagram of this laser system.

Figure 5 shows the experimental setup of the power controllable gain-switched dual-waveband Ho^3+^-doped ZBLAN fiber laser. A simplified energy level diagram is shown in the insert of Fig. 5 to illustrate the dual-waveband gain-switched transitions. The Ho^3+^ ions in the ^5^I_8_ level are pumped to the ^5^I_6_ level by the 1150 nm CW laser, which ensures the ~ 3 μm transition. Simultaneously, the 1950 nm pulsed laser periodically excites part of Ho^3+^ ions from the ^5^I_8_ level to the ^5^I_7_ level (common energy level, i.e., the upper and lower level of the ~ 2.1 μm and ~ 3 μm transitions, respectively) and acts as a modulator to induce the dual-waveband gain-switching. The 3.5-m-long double-clad Ho^3+^-doped ZBLAN fiber (FiberLabs, Japan) with a dopant concentration of 1.2 mol. % serves as the gain fiber. The absorption coefficients were measured to be 0.28 m^-1^ and 0.6 m^-1^ using cutback measurements, which could provide 62.5% and 87.8% pump absorption efficiencies for the 1150 nm and 1950 nm pump sources, respectively. The fiber has a core diameter of 10 μm with a numerical aperture (NA) of 0.16, and a D-shaped inner cladding with a circular cross section of 125-μm diameter and a NA of 0.5. Both ends of the gain fiber were cut perpendicularly. The fiber end nearest the 1150 nm CW pump source provides ~ 4% Fresnel reflection as the output coupler. The other end of the gain fiber is terminated by a dichroic mirror (DM3) as the cavity feedback, which has a high transmittance (HT) of 85% at 1950 nm and a high reflectance (HR) of > 95% above 2.1 μm. The 1150 nm CW laser is provided by two commercially available LDs (Eagleyard Photonics Berlin). The beams are polarization combined through a polarizing beam splitter (PBS) and launched into the gain fiber by focusing with a plano-convex CaF_2_ lens (Thorlabs, LA5315) with a coupling efficiency of 82%. The DM1 has a transmittance of 84% at 1150 nm and HR of 92% and 95% at ~ 3 μm and ~ 2.1 μm, respectively, and is placed at an angle of 45° to redirect the output beam laser away from the pump laser. The ~ 3 μm and ~ 2.1 μm output pulses are separated by DM2, which has > 93% HR at ~ 3 μm and > 90% HT at ~ 2.1 μm. The 1950 nm pulsed pump source is an in-house built Q-switched Mode-locked Tm^3+^-doped fiber laser (TDFL) with a master oscillator power amplifier system. The Q-switched envelope of the TDFL has a fixed pulse duration of 6.2 μs and repetition rate of 50 kHz. It is launched into the gain fiber with ~ 82% launching efficiency by a pair of identical CaF_2_ lens (Thorlabs, LA5315).

### Measurement method

The output ~ 3 μm and ~ 2.1 μm gain-switched pulse trains were detected by an InAs detector (Judson, J12-18C-R01M) and an InGaAs detector (EOT ET-5000F), respectively, and recorded by a 500-MHz bandwidth digital oscilloscope (RIGOL, China, DS4054, 500 MHz, 4GSa/s). The optical spectra of the ~ 3 μm and ~ 2.1 μm pulsed lasers were captured by a monochromator using a nitrogen cooled photodiode (Princeton instrument Acton SP2300) with resolution of 0.1 nm, and an optical spectrum analyzer (Yokogawa AQ6375) with resolution of 0.05 nm, respectively. The RF spectra of the ~ 3 μm and ~ 2.1 μm pulsed lasers were measured by a RF spectrum analyzer (YIAI, China, AV4033A, 30 Hz–18 GHz).
